# Aptamer Antagonists of Myelin-Derived Inhibitors Promote Axon Growth

**DOI:** 10.1371/journal.pone.0009726

**Published:** 2010-03-16

**Authors:** Yuxuan Wang, Zin Z. Khaing, Na Li, Brad Hall, Christine E. Schmidt, Andrew D. Ellington

**Affiliations:** 1 Department of Chemistry and Biochemistry, University of Texas at Austin, Austin, Texas, United States of America; 2 Department of Biomedical Engineering, University of Texas at Austin, Austin, Texas, United States of America; 3 Department of Chemical Engineering, Cockrell School of Engineering, University of Texas at Austin, Austin, Texas, United States of America; University of Oulu, Germany

## Abstract

Myelin of the adult central nervous system (CNS) is one of the major sources of inhibitors of axon regeneration following injury. The three known myelin-derived inhibitors (Nogo, MAG, and OMgp) bind with high affinity to the Nogo-66 receptor (NgR) on axons and limit neurite outgrowth. Here we show that RNA aptamers can be generated that bind with high affinity to NgR, compete with myelin-derived inhibitors for binding to NgR, and promote axon elongation of neurons *in vitro* even in the presence of these inhibitors. Aptamers may have key advantages over protein antagonists, including low immunogenicity and the possibility of ready modification during chemical synthesis for stability, signaling, or immobilization. This first demonstration that aptamers can directly influence neuronal function suggests that aptamers may prove useful for not only healing spinal cord and other neuronal damage, but may be more generally useful as neuromodulators.

## Introduction

Patients with spinal cord injury suffer from permanent functional deficits and paralysis due to the limited capacity of axons to regenerate. Unlike their counterparts in the peripheral nervous system (PNS), damaged axons in the central nervous system (CNS) do not regenerate spontaneously because of an inhibitory environment. Studies have shown that CNS myelin is a major source of inhibition to axon regeneration [1]–[3]. Trauma to the CNS can result in major disruptions in white matter tracts, including breakdown of myelin sheaths. Products of this myelin breakdown come in contact with the surfaces of severed axons and inhibit regeneration. The three known major myelin-derived inhibitors are Nogo-A, myelin-associated glycoprotein (MAG), and oligodendrocyte myelin glycoprotein (OMgp). All three bind with high affinity to the Nogo-66 receptor (NgR) on axonal surfaces [1]–[3]. Enzymatic cleavage of NgR confirms this effect, in that it increases axon regeneration [1]. It was recently shown that phosphorylation of NgR by casein kinase II also inhibits binding of the myelin-associated proteins and promotes regeneration [4]. Because NgR is a GPI-linked receptor and lacks an intracellular signaling domain, it relies on the transmembrane co-receptor, p75, to transduce the inhibitory signal. The final step in the signaling pathway is the activation of RhoA, a small GTPase that regulates actin polymerization and inhibits axonal elongation in its active form. Nogo-A, MAG, and OMgp activate RhoA through the NgR/p75 receptor complex, and this NgR/p75-complex/RhoA pathway is postulated to be responsible for the inhibitory signals that prevent axon regeneration [5].

Recent pharmacological methods to overcome CNS myelin inhibition involved the use of an anti-Nogo antibody [6], [7], RhoA inhibitors [8], [9], a NgR antagonist peptide [10], and soluble NgR [11].

There are potential problems with these inhibitors as therapeutic agents. For example, the direct blockade of RhoA with an inhibitor may disrupt other, crucial Rho-related cellular activities. In contrast, the anti-Nogo antibodies are only specific for Nogo and do not disrupt MAG or OMgp action. Because of this, it may be useful to identify high affinity inhibitors that more generally interact with the surface of NgR.

Aptamers are single-stranded oligonucleotides that fold into unique three-dimensional structures, allowing them to bind to protein targets with high affinity and specificity. They are an alternative to therapeutic antibodies but can be chemically synthesized in a cell-free system. Furthermore, aptamers have a number of advantages over peptide and protein antagonists, including their relatively low cost of production, ease of GMP manufacture, and the simplicity with which they can be modified for stability, signaling, and immobilization [12]–[15]. Studies have shown that aptamers have no or low immunogenicity, and are generally non-toxic [16], [17], which is a great advantage in comparison to antibodies given the length of treatment period required for spinal cord injuries. Because of this aptamers are seeing increasing clinical use. Macugen, a pegylated 2-fluoro pyrimidine RNA aptamer and a potent inhibitor of the angiogenic regulatory protein, VEGF(165) [18]–[20], was approved by the FDA for treatment of neovascular age-related macular degeneration in 2004.

Aptamers have previously been used to investigate neurological disorders, such as Alzheimer's [21], [22], multiple sclerosis [23], [24], and myasthenia gravis [25], [26]. For example, an aptamer was selected against the 40 amino-acid beta-amyloid peptide and was shown to bind fibrils consisting of the peptide [22]. But no functional data regarding fibril dissociation or reduction has been reported. Similarly, aptamers have been used to target myasthenia gravis, which is a neuromuscular disorder resulting from antibody-mediated autoimmune response to the nicotinic acetylcholine receptor (AChR). A 2′-amino-modified aptamer was isolated against Mab198, a monoclonal antibody that recognizes the major immunogenic epitope on human AchR [25]. The aptamer protected AChR from antoantibodies found in patients with myasthenia gravis. A later selection yielded a 2′-fluoropyrimidine-modified aptamer, which offered even greater protection [26]. However, in these instances aptamers have primarily been used to treat disorders, rather than to modulate normal neuronal function.

Here, we selected RNA aptamers that bind to NgR with high specificity and affinity. Most importantly, these aptamers were shown to compete with Nogo, MAG, and OMgp for binding to NgR. Neurite outgrowth assays demonstrated that these aptamers can reverse the effect of these inhibitors *in vitro*. These are the first aptamers to modulate neuronal growth.

## Results

### Selection of Anti-NgR aptamers

A RNA pool containing 50 randomized positions (R50) flanked by two constant, primer-binding regions was used as a starting point for selection. The protein target was a fusion of the extracellular domain of rat NgR and the constant region of human IgG. In each round of selection, RNA-binding species were separated from weak or non-binding species by passing the protein:nucleic acid complexes through a modified nitrocellulose filter. Captured species were amplified via reverse transcription and PCR. Negative selections were carried out to remove aptamers that bound species (the filter, IgG, and BSA) other than the target. Iterative rounds of selection and amplification were performed until the target-binding aptamers dominated the selected population.

Binding progressively increased and saturated at round 10. The selected population showed good specificity for NgR (36% RNA binding at 1 µM protein concentration), with little binding to BSA, IgG, or the filter (<1% RNA binding at 1 µM protein concentration). The population was cloned and sequenced, and a best-binding, dominant aptamer (Clone 6), was identified. The K_d_ of Clone 6 was estimated to be around several hundred nM using a three point binding assay (data not shown), which we deemed not sufficiently low to compete with endogenous inhibitors. To further improve affinity, additional variants were selected from a partially randomized pool based on Clone 6 that contained 70% wild-type and 30% non-wild type nucleotides. This level of mutagenesis should allow the accumulation of not only additional functional residues, but also changes in or additions to base-paired structures. In addition, a new selection was carried out with a slightly longer pool (N62) in order to identify aptamers that might cover larger swaths of the NgR surface and thereby prevent binding of multiple inhibitors.

At the conclusion of both selections, 56 clones were sequenced: 28 from the Clone 6 doped re-selection, and 28 from the N62 selection. As expected, the best ligands for NgR were present multiple times in the selected populations (**Figure S1**). However, no strong consensus sequences or motifs emerged from the selections. The diversity of aptamer sequences could mean that the selected aptamers interact in several different ways with NgR, a result that is consistent with the fact that very different myelin-derived inhibitors all bind to NgR.

The aptamers (Clones 40, 79, 83, 110, and 152) that were used for *in vitro* studies were chosen based both on affinity and on their ability to bind to non-overlapping sites. The identified aptamers bound NgR with K_d_ values from 21 to 61 nM (**Table 1** **; Figure S2**). Competition assays between aptamers were carried out in order to determine whether they bound to the same, different, or overlapping epitopes (**Figure S3**). For example, Clone 40 bound to a similar set of sites as Clones 29 and 39, but with higher affinity. In contrast, Clones 110 and 152 showed little ability to compete with one another, and thus likely bound very different epitopes. The fact that the aptamers seemed to bind to non-overlapping sites on NgR is consistent with the selection of multiple, different aptamers from both the original and doped sequence selections.

**Table 1:** The sequences of the random regions of the aptamers tested in neurite outgrowth assays, maximum percent binding (B_max_) in our standard assay, and dissociation constants (K_d_).

Clone	Sequence	B_max_(%)	K_d_(nM)
40	ATAACACGACATCCATATGTCAGTGGTCTGTGTACTTACACGGTATTCGA	81±7	25±8
79	ACTACACGAGGACCTACGACTACTACATTATGCCAACCGGTCTTGCTTCGACACAGATACCTC	85±8	60±16
83	TTGCACAAGATACGGCTACCTGTATGCGGCAATCGGCATTAAATCTATCTAAGCCAGCAGTAAC	65±3	21±4
110	ATAACACGACATCCATATGTCAGTGGTCTGTGTACGTACACGGTATTCGC	95±8	50±13
152	GGCGATAGTTTCTATAGCAAGGTACAGCATTCTCTCTCCCTATAGAACCAATCCAGTACTAGC	84±8	61±16

### Aptamers generally block inhibitor binding to NgR

**Figure 1:**
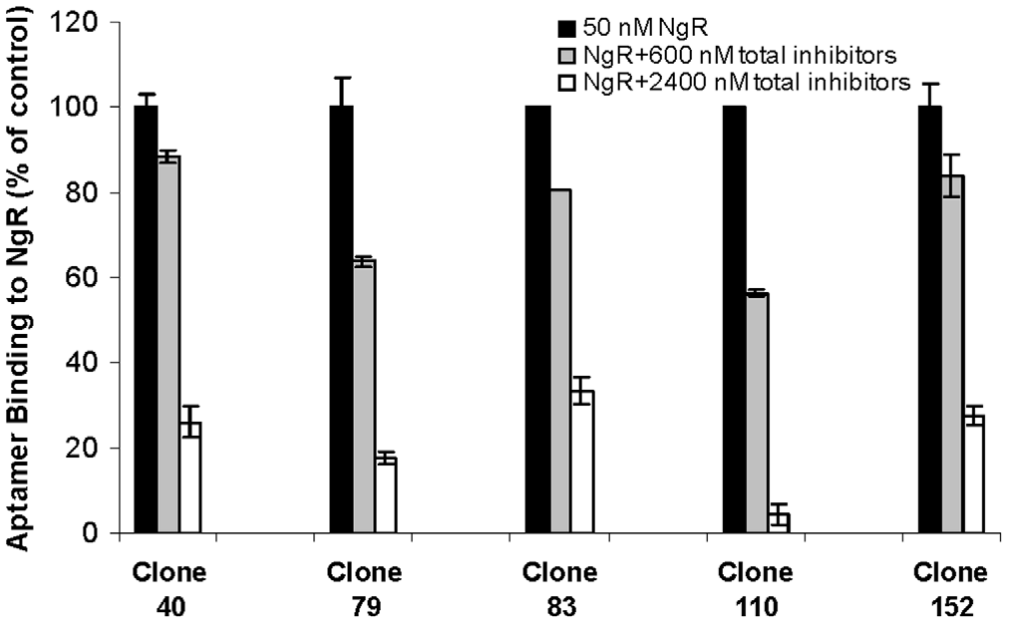
Competition assay between aptamers and myelin-derived inhibitors. The three myelin-derived inhibitors (Nogo, MAG, OMgp) at excess concentrations (200 or 800 nM for each of the three inhibitors, implying 600 and 2400 nM total inhibitor concentrations) reduced aptamer (5 nM) binding to NgR (50 nM) in a standard assay. Results were normalized to the aptamer alone controls. Binding data is the average of two determinations.

The predicted protein structure of NgR includes a signal sequence, eight central leucine-rich repeats (LRRs) flanked by a cysteine-rich C-terminal subdomian (LRRCT) and a smaller leucine-rich N-terminal subdomain (LRRNT) [1], [27]. Studies suggest that all three inhibitors (MAG, Nogo, and OMgp) bind overlapping sites in the LRR repeat domains [2], [28]. To determine whether or not the aptamers could more generally interfere with inhibitor binding, we assayed five of the best binding aptamers in competition with Nogo, MAG, and OMgp. Remarkably, aptamers blocked more than one inhibitor simultaneously, and a modest diminution of aptamer binding (3–10 fold) was only seen at the highest total inhibitor concentration (2400 nM) (**Figure 1**). As might be expected, there was a direct correlation between the affinity of the aptamers for NgR and the ability of the aptamers to compete with inhibitors for binding. The aptamers with the lowest binding constants, Clone 40 and Clone 83, also showed the lowest decrease in binding in the presence of the inhibitors.

### Aptamers localize to NgR-expressing neuronal and non-neuronal cell surfaces

**Figure 2:**
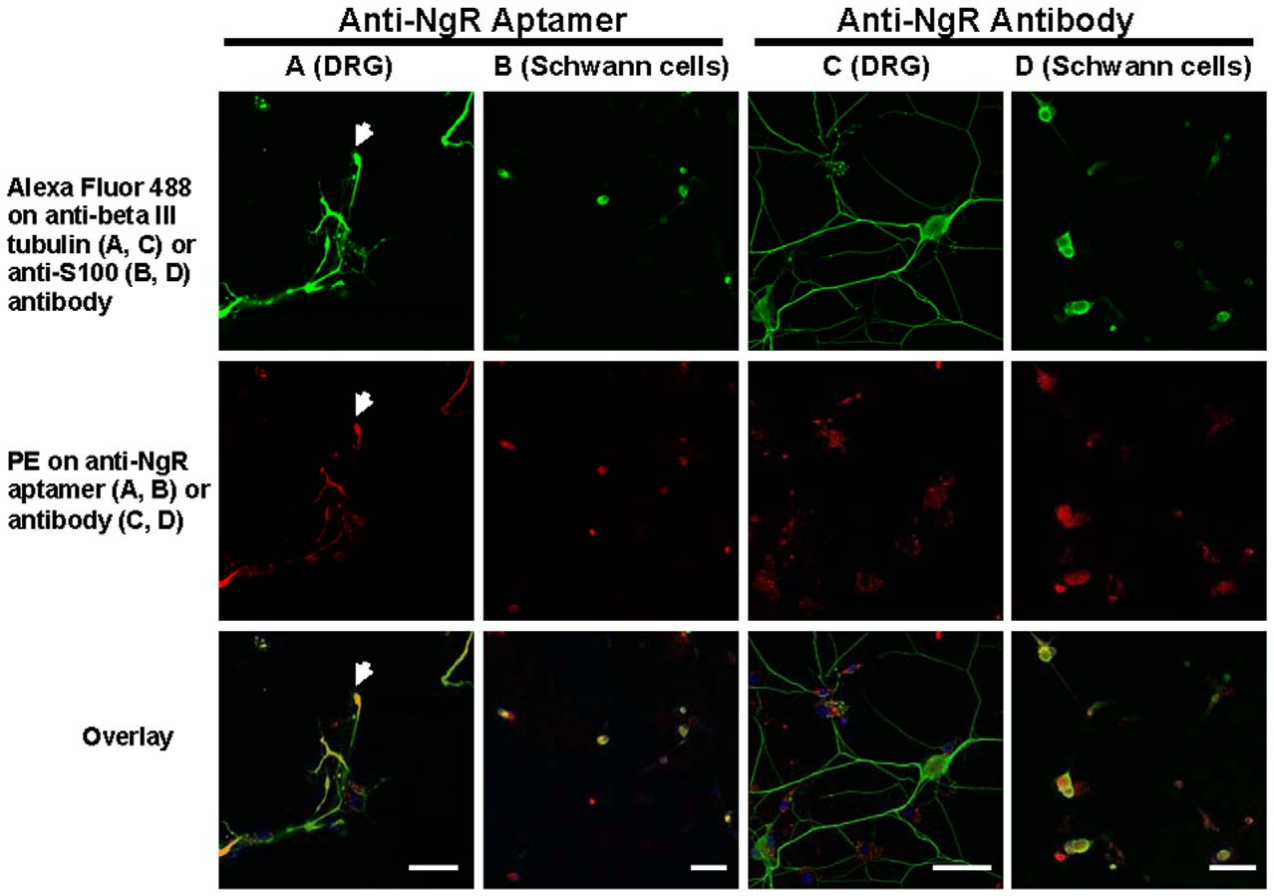
NgR expression in dorsal root ganglia and Schwann cells. DRG neurons in primary cultures were labeled using an anti-beta III tubulin antibody (green; columns A, C). Schwann cells were labeled using an anti-S100 antibody (green; columns B, D). Binding of a biotinylated anti-NgR aptamer (Clone 40) was detected with PE-labeled streptavidin (red; A, B). Binding of a biotinylated anti-NgR antibody was also detected with PE-labeled streptavidin (red; C, D). Yellow in the overlay indicates the overlap of green and red stains. Arrows in Column A indicate the position of a growth cone in a DRG neuron, and the overlay suggests localized expression of NgR. Scale bars: 25 µm (A); 50 µm (B-D).

Microscopy confirmed the binding of fluorescently-labeled aptamers to dissociated dorsal root ganglia (DRG), which have previously been shown to express NgR on their surfaces [29]. After staining with a neuron-specific anti-beta III tubulin antibody, we found NgR expression in non-neuronal cell types in addition to DRG neurons (data not shown). To determine the nature of these non-neuronal, NgR-expressing cells, we stained the culture with an anti-S100 antibody, which identifies Schwann cells. The co-localization of the anti-NgR aptamers with the anti-S100 antibody suggested that NgR is expressed on Schwann cells. This discovery was further confirmed with an anti-NgR antibody, which again bound both neurons and Schwann cells (**Figure 2**). There was little to no binding to a cell line (A431) that lacked NgR, although the 431 cells were readily stained with a positive control, an anti-EGFR aptamer (**Figure S4**).

### Disinhibition of neurite outgrowth

To the extent that aptamers can compete with the inhibitors Nogo, MAG, and OMgp for binding to NgR, they were likely to show one of two biological effects: potentiation of neurite outgrowth (due to inhibition of the inhibitors) or inhibition of neurite outgrowth (due to mimicking of the inhibitors). To choose between these hypotheses, dorsal root ganglia (DRG) were isolated from P1-P3 rats and dissociated into single cells. The primary cultures included neurons and non-neuronal cells, such as Schwann cells. Throughout the study, the mitotic inhibitor 5-fluoro-2′-deoxyuridine (5-FDU) was added to arrest proliferation of non-neuronal cells.

**Figure 3:**
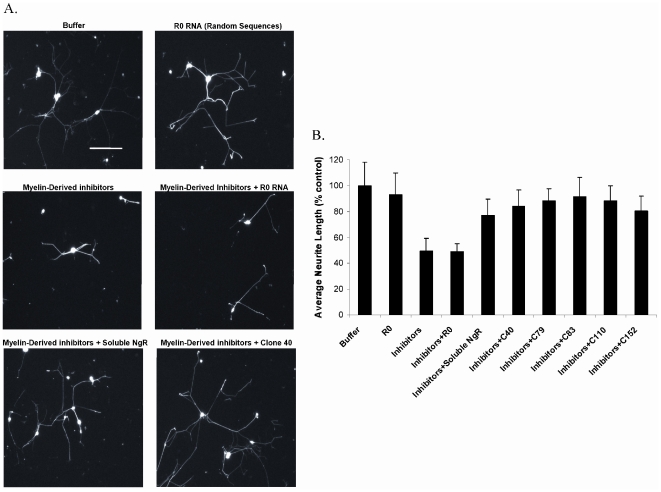
Neurite outgrowth assays with dorsal root ganglia. The myelin-derived inhibitors (Nogo, MAG, and OMgp) were added at 300 nM total (100 nM each). Aptamers (Clones 40, 79, 83, 110, and 152) were added at a concentration of 10 µM. (A) Visualization of neurite lengths. DRGs were stained with a neuron-specific anti-beta III tubulin antibody. The pictures shown are representative examples. Scale bar, 200 µm. (B) Average neurite lengths. Data was collected over 200–500 neurons per condition, and means + standard deviations are reported. Binding was normalized to a buffer alone control.

In accordance with previous studies [28], [30]–[33], the three myelin-derived inhibitors reduced axon elongation, as measured by the length of neurites formed in culture (p<0.0001). Approximately 200–500 cells were measured for each condition in triplicate (**Figure 3**). In contrast, the addition of aptamers increased growth significantly relative to the inhibitors-only control (p<0.001). Recovery of growth ranged 80–91% relative to the no-inhibitor control, almost a two-fold increase from full inhibition. Clone 83, the aptamer with the lowest binding constant, resulted in the highest reversal of inhibition among the aptamers, although at the concentrations assayed all of the aptamers showed similar effects on neurite outgrowth. In contrast, the negative control, random RNA molecules at the same concentration, did not affect axon elongation. Because all RNA samples were prepared at the same time using the same reagents and methods, this shows that the aptamers promoted neurite outgrowth based on their particular sequences and structures alone.

Soluble NgR(310)ecto was used as a positive control and could also compete with inhibitors and increase neurite outgrowth [11]. The soluble receptor at 1 µM, a concentration more than 3-fold that of the inhibitors, was not as effective as the aptamers in reversing the inhibition. A further disadvantage of the soluble NgR(310)ecto is that it only sequesters inhibitors in a one-to-one ratio whereas one aptamer could compete with the binding of multiple inhibitors (**Figure S5**).

## Discussion

The myelin-associated inhibition of axon regeneration presents a major barrier to recovery from central nervous system injury. Here, we have shown that RNA aptamers can be selected to bind the Nogo-66 receptor (NgR) and compete with myelin-derived inhibitors of axon regeneration for binding to the receptor. Furthermore, neurite outgrowth assays demonstrated that these aptamers can reverse the effect of these inhibitors *in vitro*.

The selected aptamers have binding affinities (21 to 61 nM) lower or comparable to other protein antagonists. The anti-Nogo antibodies bind to Nogo with K_d_ values of 8 µM and 1 µM, for the wild-type and engineered II.1.8 mutant, respectively [34]; while the NgR antagonist peptide NEP 1–40 shows half-maximum inhibition at 50 nM [10]. Furthermore, in contrast to existing antagonists, these aptamers blocked more than one inhibitor simultaneously and could more generally interfere with inhibitor binding. The NEP1–40 peptide significantly, but only partially, blocks myelin inhibition [10]. This is because the peptide antagonist only blocks Nogo-66 mediated activity but not that of MAG [35], [36]. Similarly, a disadvantage of the soluble NgR(310)ecto is that it only sequesters inhibitors in a one-to-one ratio whereas one aptamer could compete with the binding of multiple inhibitors. Given that all three inhibitors (MAG, Nogo, and OMgp) bind overlapping sites in the leucine rich repeat (LRR) domains of NgR [2], [3], molecules that generally interfere with the binding of all three inhibitors would have the best efficacy.

Using these aptamers as detection tools, we unexpectedly found the expression of NgR on Schwann cells. The localization of both the anti-NgR aptamers and antibody to Schwann cell surface led us to speculate the role of NgR on these cells. Schwann cells form the basis for nerve regeneration and repair in the PNS. Upon losing contact with axons such as following injury, mature Schwann cells undergo developmental regression and proliferation to provide an environment inductive to axonal re-growth [37]. However, Schwann cells were previously reported not to express either Nogo or its receptor via *in situ* hybridization [38]. The difference between the previous study and our own findings might be the age-dependent expression of NgR in Schwann cells. The rat pups used in our experiments were newborns; whereas the mice pups used in the previous study were postnatal day 7. NgR expression might be downregulated as the animals reach adulthood, as previously observed in the spinal cord for humans and mice [38].

Studies have shown that the lack of regeneration in the CNS results from a hostile environment. For example, injured CNS axons can extend over long distances in the presence of a peripheral nerve graft [39]. Given that PNS is significantly more permissive for growth relative to the CNS and that Schwann cells have a role in inducing axonal re-growth in the PNS, it is possible that these surface receptors act as competitive binders for myelin-derived inhibitors that might otherwise come in contact with growing axons. Indeed, given the larger number of Schwann cells, it is possible that these cells act as a ‘buffer’ for the varying amounts of myelin-derived inhibitors that may be released. It is also interesting that these supporting cells express both NgR and myelin-proteins, such as MAG, but not Nogo and OMgp [40]. This suggests an interesting scenario in which early Schwann cells can express both inhibitors of neurite growth and proteins that will bind the inhibitors, allowing the concentrations of these inhibitors to be very finely tuned in order to fine-tune neurite outgrowth over time.

Our study shows the potential use of aptamers as a therapeutic to overcome the myelin-associated inhibition to regeneration. The aptamers prove to be better growth promoters than other, protein-based compounds that have previously been assayed, and may offer a novel therapeutic modality for neural regeneration. That said, the aptamers did not compete with peptides as well as their affinity constants might have indicated. The selection of aptamers that include modified nucleotides would significantly improve the ability to compete in serum and eventually in animal models, and we are now pursuing these studies. Most importantly, this work shows that aptamers can be valuable tools not only in neuopathologies, but also in modulating and redefining normal neuronal architectures.

Other than its function in restricting neurite outgrowth, NgR also has an apparent role in preventing NGF-stimulated p75NTR-dependent motor neuron death as recently shown [41]. Peptides derived from one of its ligands, Nogo, exert neuroprotective effects through NgR binding. It would be interesting to study the effect of these aptamers to determine whether or not they can both prevent motor neuron death and promote their axonal elongation.

## Materials and Methods

### *In Vitro* Selection

Aptamers against NgR were generated by *in vitro* selection, starting from synthetic DNA libraries. For the R50 pool, an oligonucleotide containing a 50-nucleotide random region (N50) flanked by constant regions were synthesized: 5′- GATAATACGACTCACTATAGGGTTTACCTAGGTGTAGATGCT-N50-AAGTGACGTCTGAACTGCTTCGAA-3′. The underlined portion is the T7 RNA polymerase promoter region. Similarly, the N62 pool consisted of a 62-nucleotide (N62) region flanked by a different pair of primers: 5′ - GATAATACGACTCACTATAGGCGCTCCGACCTTAGTCTCTG-N62-GAACCGTGTAGCACAGCAGA - 3.′ Following *in vitro* transcription by T7 RNA polymerase (Epicenter Technologies, Madison, WI), the derived RNA library was purified on an 8% denaturing polyacrylamide gel, then eluted and precipitated. In order to eliminate aptamers from the population that bound to either the filter or the Fc region, an initial negative selection was carried out. The RNA population (∼200 pmol, 10^14^ sequences) was incubated with 200 pmol of recombinant human IgG Fc (R&D Systems, Minneapolis, MN) and 0.01% BSA for 30 mins at 37°C. Bound species were separated from unbound on a modified cellulose filter (Millipore, Bedford, MA). The molecules that passed through the filter were collected and then incubated with recombinant rat Nogo receptor/Fc chimera (R&D Systems, Minneapolis, MN) in selection buffer (PBS, pH 7.4, 3 mM MgCl_2_) for 30 mins. Since the rat sequence (SwissProt entry Q99M75) of the immunogen peptide and the mouse sequence (SwissProt entry Q99PI8) are 100% identical, the difference in species does not present a problem for later neurite outgrowth assays using rat neurons. Separation through a modified cellulose filter again trapped proteins and bound RNAs. After washing with 600 µL Selection Buffer, RNA molecules were eluted from the filter in 200 nM NaCl, 50 mM Tris-HCl, pH 7.4, 10 mM DTT, and 6 mM MgCl_2_, and amplified by reverse transcription and the polymerase chain reaction (Invitrogen, Carlsbad, CA). An aliquot of the amplified DNA template (∼1 µg or 20 pmol, roughly one-sixth of total amount of amplicon produced) was used in a transcription reaction to generate an RNA pool for the subsequent round of selection. Iterative rounds of selection and amplification were performed until the target-binding aptamers dominated the library population. In later rounds, the protein: RNA ratio, the MgCl_2_ concentration, and the incubation time during the binding reaction were altered to increase stringency. The protein:RNA ration was 1∶1 for the first two rounds, and 1∶2 for subsequent rounds (protein was kept constant at 200 pmol, and RNA was varied from 200 to 400 pmol). The MgCl_2_ concentration was 5 mM for the first three rounds and 3 mM for the subsequent rounds. The incubation time was 30 mins for the first three rounds and 20 mins for later rounds. Following sequencing, the MEME server [http://meme.sdsc.edu] was used to find potential sequence motifs.

Selections to optimize Clone 6, the dominant aptamer from the initial selection (**Figure S1**), were performed in a similar manner. The sequence for the partially randomized pool based on Clone 6 was 5′GGGTTTACCTAGGTGTAGATGCT*CTCTCTATTTCATTCGTAGGCTATTGGTGCCGCAAATACTAGCTTTACGA*AAGTGACGTCTGAACTGCTTCGAAAA - 3′, where the italicized residues indicate positions that were synthesized at 70% wild-type and 30% non-wild-type nucleotides.

### Binding Assays

The selected population was characterized for binding to NgR using a filter-binding assay similar to that used for the selection. The aptamers were radioactively labeled in a transcription reaction using [α-^32^P]ATP (3000 ci/mmol, 10 mCi/ml, Perkin Elmer, Waltham, MA). The labeled RNA was then incubated with 12.5, 18.75, 25, 50, 75, 150, 300, and 500 nM of the target protein (NgR/FC chimera). To separate the RNA bound to protein from free RNA, the reaction was passed through a nitrocellulose (top) then a nylon (bottom) filter (Schleicher & Schuell, Keene, NH) on a vacuum manifold. The nitrocellulose captured the RNA:protein complexes, while the nylon captured free RNA. After washing with Selection Buffer (600 µL), the filters were dried and exposed to a PhosphorImager plate (GE Healthcare, Piscataway, NJ) to quantitiate the amount of retained radioactivity. The fraction of a given RNA (population or aptamer) that bound the target proteins was calculated by comparing the counts retained on the filter with the total number of counts in the original RNA. Binding to IgG and BSA were assayed similarly. The maximum binding (B_max_) and dissociation constants (K_d_) were fit using the program SigmaPlot (Chicago, IL). The K_d_ curves were plotted in SigmaPlot according to the following equation:
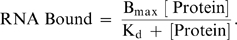



### Competition Binding Assays

Competition between aptamers and myelin-derived inhibitors was assayed by measuring the reduction in aptamer binding in the presence of the inhibitors. The aptamers were again radioactively labeled during transcription as described above. The NgR/Fc chimera (50 nM) was incubated with Nogo, MAG, and OMgp (R&D Systems, Minneapolis, MN, each at 200 nM or 800 nM) for 5 mins. Aptamers were then introduced and incubated with the mixture for 20 mins at 37°C. The binding reaction was passed through filters on a vacuum manifold, and the filters were washed and quantified as described in the binding assays above. The fraction of RNA bound was measured. Decreases in aptamer binding due to the formation of inhibitor-NgR complexes were determined in comparison with an aptamer alone control.

### Neurite Outgrowth Assays

Dorsal root ganglia (DRG) were removed from P1-P3 rats and trimmed to remove axons for accurate measurements of re-growth in RPMI (Sigma-Aldrich, St. Louis, MO). DRGs were dissociated into single cells by incubation with 0.25% collagenase type I (Sigma-Aldrich) at 37°C for 30 mins and 0.025% trypsin (Sigma-Aldrich, St. Louis, MO) for 37°C for 10 mins. Dulbecco's Modified Eagle's Medium (Sigma-Aldrich) with 10% fetal bovine serum (Hyclone, Logan, UT) and 1% penicillin-streptomycin (Sigma-Aldrich) was added to dissociated cells, and the cells were plated in 24-well plates coated with 50 µg poly-L-lysine/mL (Sigma-Aldrich, St. Louis, MO) and 10 µg laminin/mL (Trevigen, Gaithersburg, MD) at a density of 50,000 cells/well. At the time of plating, 30 nM of 5-fluoro-2′-deoxyuridine (Sigma-Aldrich) was added to arrest proliferation of non-neuronal cell types. The three myelin-derived inhibitors (each at a final concentration 100 nM), RNAse inhibitors at 1.6 Units/µl (Ambion, Austin, TX), and a given RNA (final concentration 10 µM) were added an hour after plating (to minimize interference with cell adherence). After 24 hours of incubation, the cells were fixed in 4% paraformaldehyde, immunostained with a neuron-specific anti-β-tubulin III antibody (Abcam, Cambridge, MA) and an Alexa Fluor 488-labeled goat anti-rabbit IgG (Invitrogen, Carlsbad, CA) as the secondary antibody. To assay the impact of compounds on neurite outgrowth, the lengths of individual immunostained neurites were measured. The cell density and growth time were optimized to ensure that neurons grew in relative isolation from each other and that neurites from nearby cells did not fasciculate. An inverted fluorescence microscope (IX-70, Olympus) was used to visualize the cells, and images from the microscope were acquired using a color CCD video camera (Optronics Magna-Fire, model S60800). Quantification of neurite outgrowth was performed over a sample of 200–500 neurons per condition using Leica QWIN Image Analysis software (Leica Microsystems, Bannockburn, IL). Gray image processing was first used to identify all anti-β-tubulin III-stained neurons. Cell bodies were subsequently removed using BINARY EDIT functions. The curve lengths (defined as the length of the longest side of a rectangle having the same area and perimeter as the measured feature) of the remaining neurites on 200–500 neurons were measured and averaged. The significance of the collected data was assessed using a pairwise t-test with Bonferroni correction (**Table S1**).

### Aptamer Localization

To better label the aptamers, a 3′ extension was added by PCR. The extensions were 5′ *CTGGTCATGGCGGGCATTTAATTC*TTCGAAGCAGTTCAGACGTCACTT 3′ for the R50 pool; and 5′ *CTGGTCATGGCGGGCATTTAATTC*TCTGCTGTGCTACACGGTTC 3′ for the N62 pool. The italicized portions indicate regions of complementarity to a biotinylated oligonucleotide. RNAs (1 µM) transcribed from the extended templates were purified and hybridized to the biotinylated oligonucleotide (5′ GAATTAAATGCCCGCCATGACCAG *3′* ) (1 µM) by heating to 70°C for 3 mins and cooling to 25°C. The RNA:DNA hybrid was further incubated with streptavidin:phycoerythrin (PE) (1 uM) to generate fluorescent complexes. Dissociated DRGs were prepared as described above. The entire aptamer:biotinylated oligonucleotide:streptavidin:PE complex was incubated with the dissociated DRG culture for 30 mins in the presence of RNAse inhibitors at 1.6 units/µl (Ambion, Austin, TX). The DRGs were then washed 3 times with selection buffer (PBS + 3 mM MgCl_2_), fixed in 4% paraformaldehyde, and immunostained with a neuron-specific anti-β-tubulin III antibody (Abcam, Cambridge, MA) or an anti-S100 antibody (Abcam, Cambridge, MA) with an Alexa Fluor 488-labeled goat anti-rabbit IgG (Invitrogen, Carlsbad, CA) as the secondary antibody. Staining with a biotinylated anti-NgR antibody (R&D Systems, Minneapolis, MN) was performed similarly, except that the antibody was conjugated to streptavidin:PE. Fluorescence images were collected using a Leica SP2 AOBS confocal microscope. The brightness and contrast of images were enhanced in Adobe Photoshop (Adobe, San Jose, CA) to optimize visualization.

## Supporting Information

Figure S1 Aptamer sequences. The random regions of aptamers from the R50 doped selection (A) and the N62 selection (B) are shown. Out of the 58 clones sequenced, there were 23 distinct sequences. Several 5-bp long motifs (colored) appeared in multiple aptamers. (*) indicates the highest-affinity binders based on a single point binding assay, many of which appeared multiple times in the cloned population. These high-affinity binders were competed with one another to identify aptamers that bound to distinct regions on NgR (Figure S3). (†) indicates the highest affinity aptamers that bound to relatively non-overlapping regions on NgR and that were further tested in neurite outgrowth assays (see also Table 1).
(0.34 MB TIF)

Figure S2 Determination of aptamer binding constants. The protein concentrations were 12.5, 18.75, 25, 50, 75, 150, 300, and 500 nM. The RNA concentration was 0.5 nM. Binding curves were fit using the program SigmaPlot to yield Kd and Bmax values.
(0.01 MB TIF)

Figure S3 Competition between aptamers for binding to NgR. The highest affinity species from the R50 doped re-selection (C29, C37, C39, C40, and C110) and the N62 selection (C79, C83, and C152) (Figure S1) were chosen to compete with one another in order to identify binders to non-overlapping regions on NgR. Black denotes the binding of the radiolabeled aptamer (10 nM) to NgR (100 nM), without competition. Binding of a radiolabeled aptamer in the presence of a cold aptamer competitor (500 nM; or a 50:1 ratio of cold:radiolabeled aptamer) is shown with a different color. Based on these results, C29 and C39 have similar competition profiles to C40, but bound with lower apparent affinity. Therefore, C29 and C39 were not further investigated. Likewise, C37 bound similarly to C152 but with lower affinity.
(5.82 MB TIF)

Figure S4 Anti-NgR aptamers are specific for neuronal cell lines. Biotinylated aptamers and antibodies were labeled using Alexa568 streptavidin. The human epithelial carcinoma tissue culture cell line A431 expresses EGFR (epidermal growth factor receptor) but not NgR. Thus an anti-NgR aptamer (Clone 40) and an anti-NgR antibody showed little binding to these cells (no bright spots, top two panels) relative to an anti-EGFR aptamer (bottom panel).
(5.82 MB TIF)

Figure S5 Competition between aptamers and individual myelin-derived inhibitors. The three myelin-derived inhibitors (Nogo, MAG, OMgp) at excess concentrations (800 nM each) were incubated with the aptamer (10 nM) and NgR (50 nM) in a standard binding assay. MAG and OMgp generally reduce aptamer binding. This suggests these inhibitors and aptamers bind to overlapping or identical sites on NgR. It should be noted that even though Nogo does not appear to compete with the aptamers, it also does not appear to be effective in reducing aptamer-stimulated neurite outgrowth (Figure 3), suggesting it may bind more weakly than aptamers to NgR.
(5.82 MB TIF)
