# Supplementary figures and images for: Aptamer Antagonists of Myelin-Derived Inhibitors Promote Axon Growth
Source: PLoS One. 2010 Mar 16;5(3):e9726. doi: 10.1371/journal.pone.0009726 (PMC2838799; doi:10.1371/journal.pone.0009726)

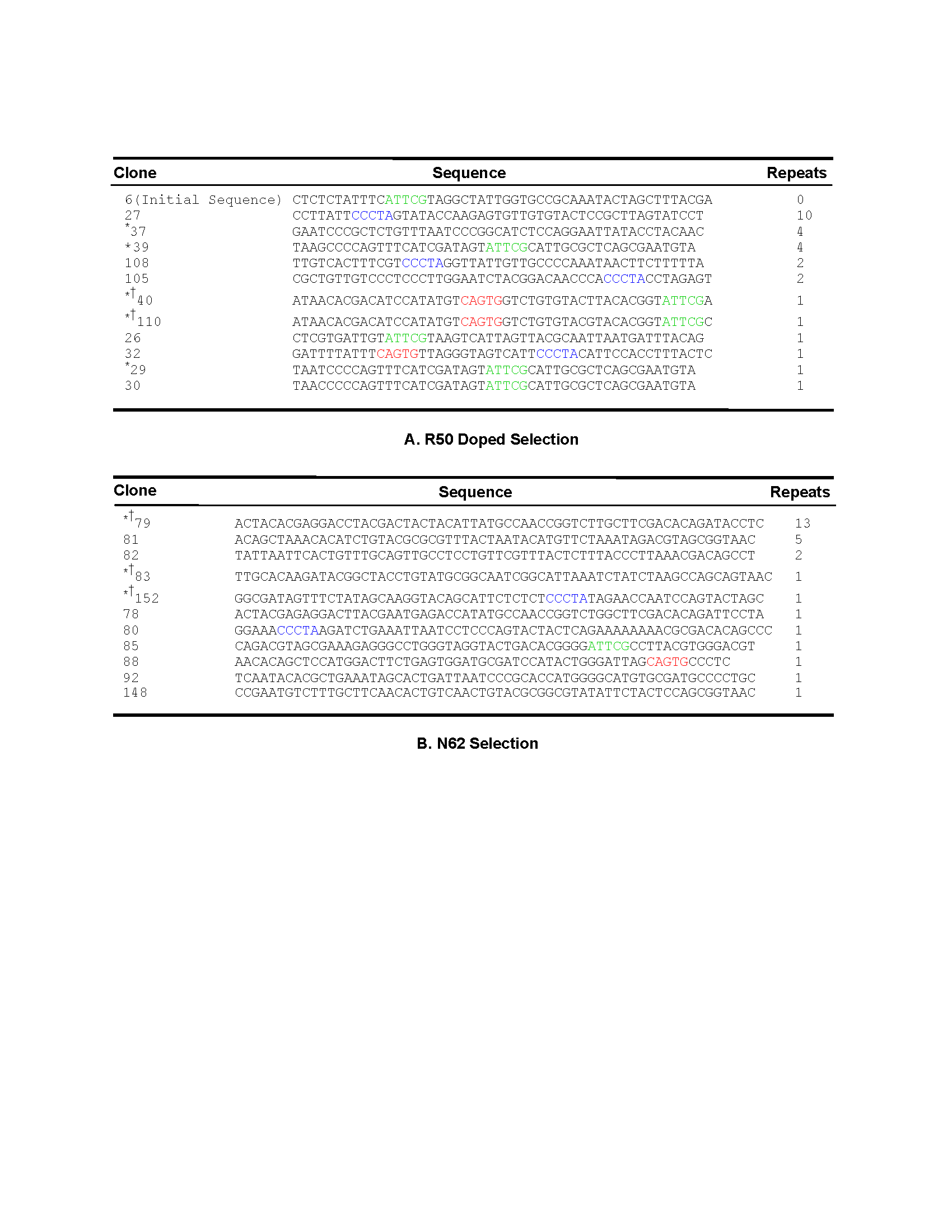

Supplement: Figure S1 — Aptamer sequences. The random regions of aptamers from the R50 doped selection (A) and the N62 selection (B) are shown. Out of the 58 clones sequenced, there were 23 distinct sequences. Several 5-bp long motifs (colored) appeared in multiple aptamers. (*) indicates the highest-affinity binders based on a single point binding assay, many of which appeared multiple times in the cloned population. These high-affinity binders were competed with one another to identify aptamers that bound to distinct regions on NgR (Figure S3). (†) indicates the highest affinity aptamers that bound to relatively non-overlapping regions on NgR and that were further tested in neurite outgrowth assays (see also Table 1). (0.34 MB TIF) [file pone.0009726.s001.tif]

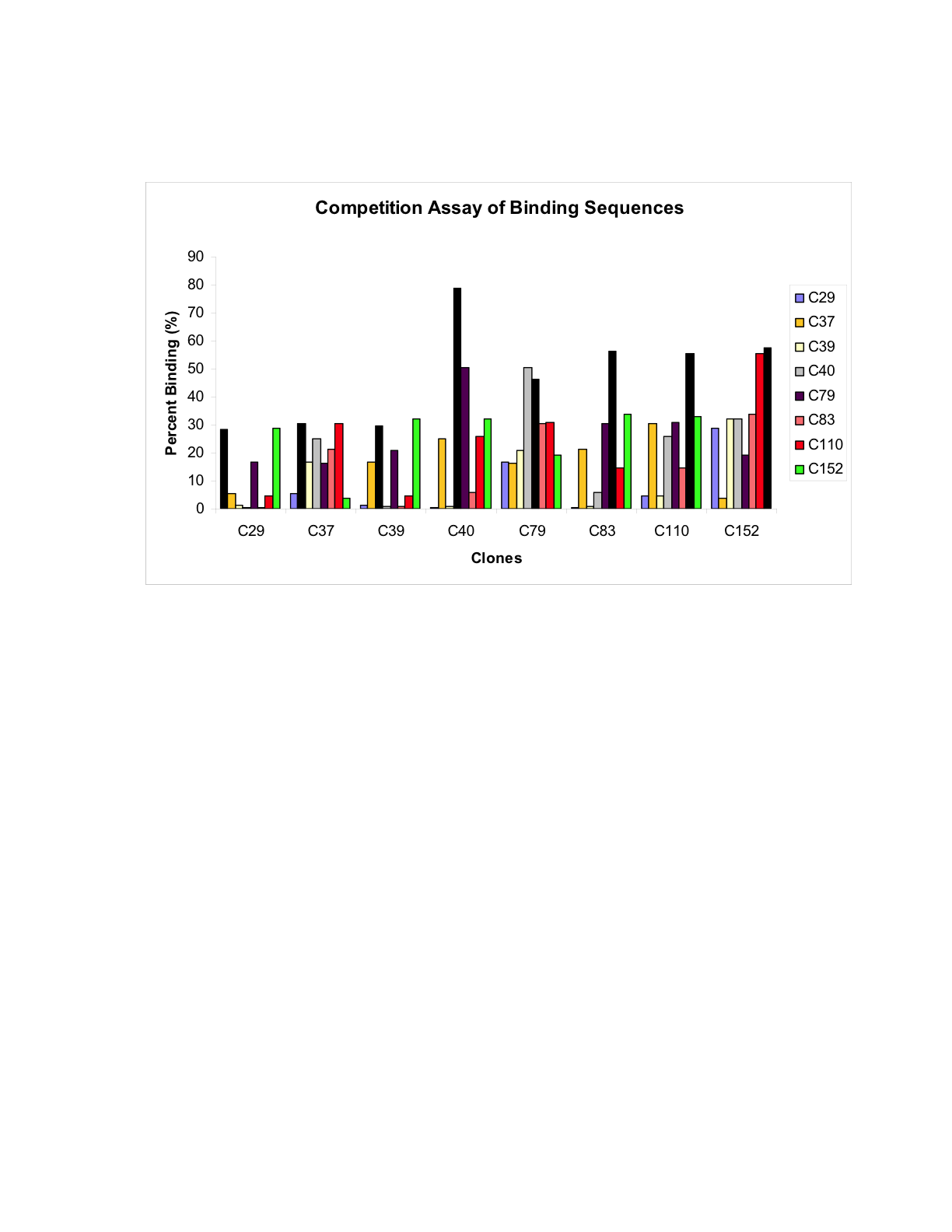

Supplement: Figure S3 — Competition between aptamers for binding to NgR. The highest affinity species from the R50 doped re-selection (C29, C37, C39, C40, and C110) and the N62 selection (C79, C83, and C152) (Figure S1) were chosen to compete with one another in order to identify binders to non-overlapping regions on NgR. Black denotes the binding of the radiolabeled aptamer (10 nM) to NgR (100 nM), without competition. Binding of a radiolabeled aptamer in the presence of a cold aptamer competitor (500 nM; or a 50:1 ratio of cold:radiolabeled aptamer) is shown with a different color. Based on these results, C29 and C39 have similar competition profiles to C40, but bound with lower apparent affinity. Therefore, C29 and C39 were not further investigated. Likewise, C37 bound similarly to C152 but with lower affinity. (5.82 MB TIF) [file pone.0009726.s003.tif]

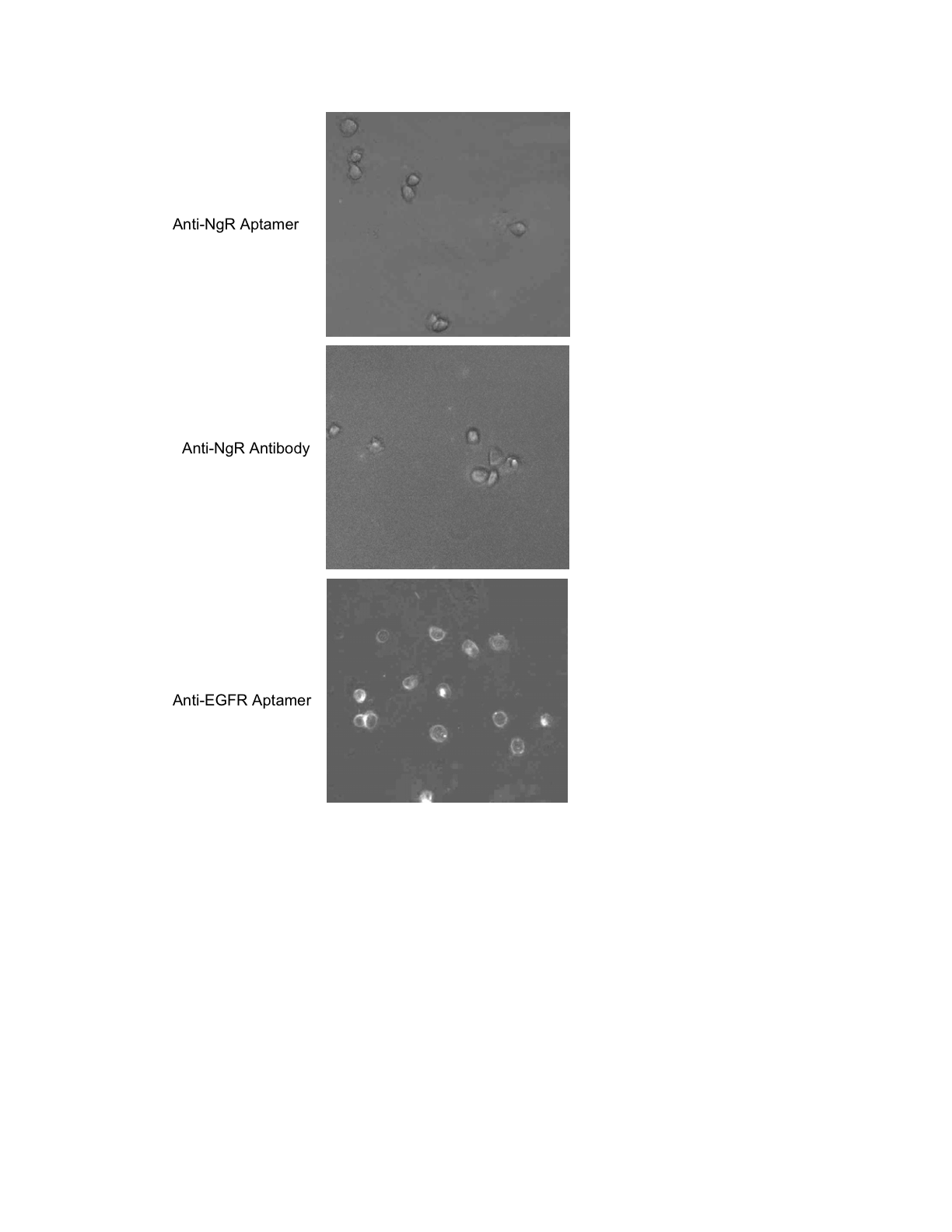

Supplement: Figure S4 — Anti-NgR aptamers are specific for neuronal cell lines. Biotinylated aptamers and antibodies were labeled using Alexa568 streptavidin. The human epithelial carcinoma tissue culture cell line A431 expresses EGFR (epidermal growth factor receptor) but not NgR. Thus an anti-NgR aptamer (Clone 40) and an anti-NgR antibody showed little binding to these cells (no bright spots, top two panels) relative to an anti-EGFR aptamer (bottom panel). (5.82 MB TIF) [file pone.0009726.s004.tif]

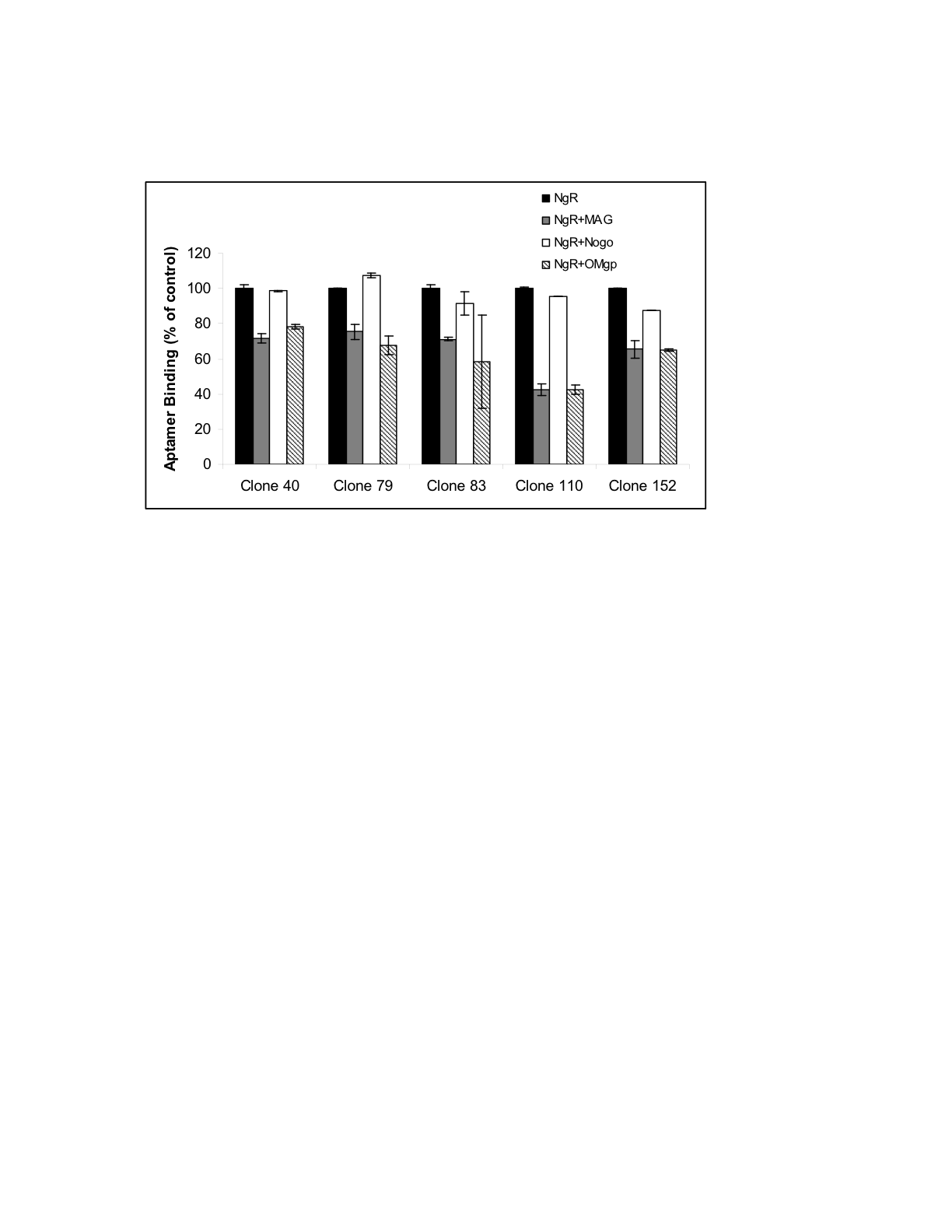

Supplement: Figure S5 — Competition between aptamers and individual myelin-derived inhibitors. The three myelin-derived inhibitors (Nogo, MAG, OMgp) at excess concentrations (800 nM each) were incubated with the aptamer (10 nM) and NgR (50 nM) in a standard binding assay. MAG and OMgp generally reduce aptamer binding. This suggests these inhibitors and aptamers bind to overlapping or identical sites on NgR. It should be noted that even though Nogo does not appear to compete with the aptamers, it also does not appear to be effective in reducing aptamer-stimulated neurite outgrowth (Figure 3), suggesting it may bind more weakly than aptamers to NgR. (5.82 MB TIF) [file pone.0009726.s005.tif]
